# WebChem Viewer: a tool for the easy dissemination of chemical and structural data sets

**DOI:** 10.1186/1471-2105-15-159

**Published:** 2014-05-23

**Authors:** Jacob D Durrant, Rommie E Amaro

**Affiliations:** 1 Department of Chemistry & Biochemistry and the National Biomedical Computation Resource, University of California, San Diego, La Jolla, CA 92093, USA

**Keywords:** Bioinformatics, Data sharing, Collaborative tools, Molecular structures, HTML

## Abstract

**Background:**

Sharing sets of chemical data (e.g., chemical properties, docking scores, etc.) among collaborators with diverse skill sets is a common task in computer-aided drug design and medicinal chemistry. The ability to associate this data with images of the relevant molecular structures greatly facilitates scientific communication. There is a need for a simple, free, open-source program that can automatically export aggregated reports of entire chemical data sets to files viewable on any computer, regardless of the operating system and without requiring the installation of additional software.

**Results:**

We here present a program called WebChem Viewer that automatically generates these types of highly portable reports. Furthermore, in designing WebChem Viewer we have also created a useful online web application for remotely generating molecular structures from SMILES strings. We encourage the direct use of this online application as well as its incorporation into other software packages.

**Conclusions:**

With these features, WebChem Viewer enables interdisciplinary collaborations that require the sharing and visualization of small molecule structures and associated sets of heterogeneous chemical data. The program is released under the FreeBSD license and can be downloaded from http://nbcr.ucsd.edu/WebChemViewer. The associated web application (called “Smiley2png 1.0”) can be accessed through freely available web services provided by the National Biomedical Computation Resource at http://nbcr.ucsd.edu.

## Background

Biological and chemical projects often generate large amounts of chemical data, ranging from percent yields to docking scores to *in vivo* drug activities. Sharing these data sets effectively is a common task that is greatly enhanced when images of the relevant molecular structures are incorporated into collaborative reports. There is a need for a simple, free, open-source program that can automatically generate data files viewable on any computer, regardless of the operating system and without requiring the installation of additional software. To this end, we have created a program called WebChem Viewer with unique capabilities currently lacking in similar software packages. The program can be downloaded from http://nbcr.ucsd.edu/WebChemViewer and is released under the FreeBSD license.

By default, WebChem Viewer inserts images of molecular structures into user-provided data sets using the Open Babel software package [1]. However, to simplify the user experience we also created an online web application (i.e., Opal service [2]) called “Smiley2png 1.0” that can generate these images remotely, thus eliminating the need for a local Open Babel installation. We encourage the use of this remote service independent of WebChem Viewer, both directly through its web interface and programmatically as a component of other software packages. The service can be accessed through the National Biomedical Computation Resource’s Web Services Opal Dashboard, which is directly linked to from the NBCR homepage at http://nbcr.ucsd.edu. Tutorials describing how to use WebChem Viewer and Simley2png can be found in the Supporting Information (Additional files 1 and 2).

## Implementation

**Figure 1:**
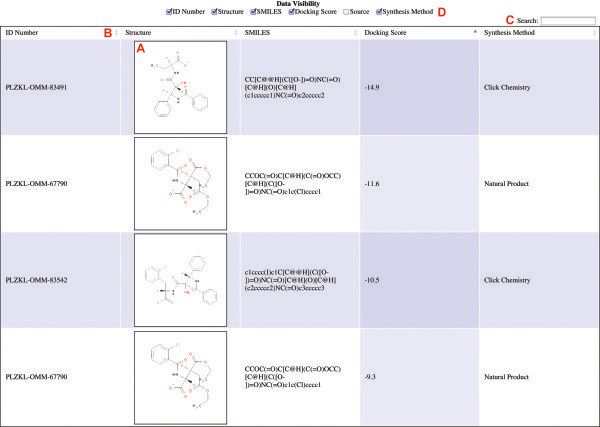
**Sample WebChem-Viewer output. A)** Two-dimensional representations of each molecule are provided by Open Babel or a remote server. **B)** The data set can be sorted by any column. **C)** The data is fully searchable. **D)** Data columns can be hidden/displayed as required.

WebChem Viewer generates HTML-formatted output that can be viewed in any modern web browser without requiring the installation of additional software or plugins. Collaborators need only open the output file in their browsers to view the chemical data sets with associated molecular representations. The data is sortable by any column (Figure 1B) and is fully searchable (Figure 1C). Data columns can also be hidden/displayed as needed (Figure 1D). These features are provided by the JQuery [3] and DataTables [4] javascript libraries, which are released under the MIT and BSD licenses, respectively, as well as by custom javascript and HTML code created by the authors. The two-dimensional molecular images included in the output are programmatically generated from user-provided SMILES strings.

To aid those generating these output files, we also kept the required dependencies of WebChem Viewer itself to a minimum. Strictly speaking, the program requires only a python interpreter. Python comes installed by default on OS X and most Linux distributions. Simple-to-use installers are available for Windows as well. We recommend the free Anaconda python distribution provided by Continuum Analytics, Inc. (http://continuum.io/downloads).

**Figure 2:**
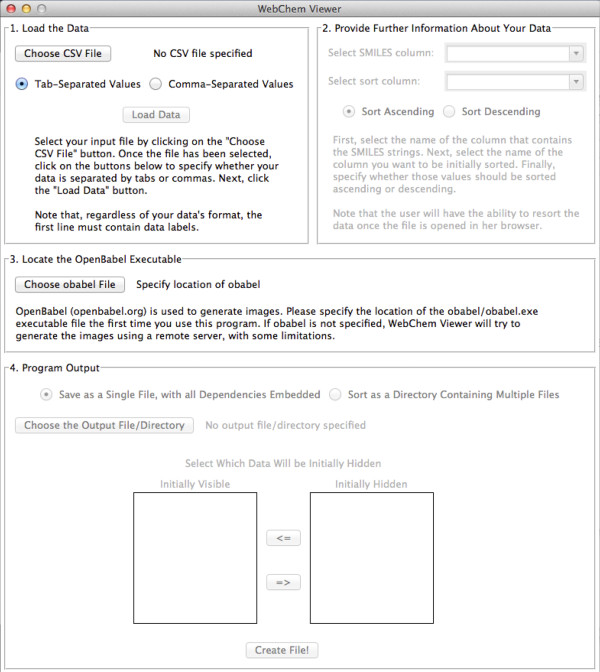
The WebChem-Viewer graphical user interface (GUI).

WebChem Viewer’s enhanced features may require some additional installations. For example, the program includes a graphical user interface (GUI, Figure 2) for those not comfortable using the command line. The GUI requires that Tkinter [5], a python binding to the Tk GUI toolkit [6], be installed. Fortunately, as Tkinter is included in the standard Windows and OS X python distributions as well as many Linux distributions, we expect the majority of users will have access to the GUI “out of the box.” Further details can be found in the Results and Discussion section below.

WebChem Viewer also uses Open Babel [1] and the accompanying Cairo 2D graphics library to generate two-dimensional molecular images from SMILES strings (Figure 1A). Most users will not need to install these programs on their machines either. WebChem Viewer automatically connects to a remote image-generating server (called “Simley2png 1.0”) if Open Babel is not available locally. If users are concerned about posting their data to a public server, or if they wish to generate more than the server’s maximum permitted number of images (currently 200), they can install Open Babel on their own machines. Versions are available for all major operating systems.

## Results and discussion

A number of programs exist for sharing tabular lists of heterogeneous molecular data with associated structures. However, frequently molecular data sets must be shared with collaborators who don’t have the required software installed on their computers; some packages are not available on all operating systems; many of the relevant tools are prohibitively expensive; and many programs, feature rich by design, are excessively complex for simple data sharing. In order to address these challenges, we have created a program called WebChem Viewer capable of organizing molecular data sets in a visual and intuitive way.

### WebChem viewer input

WebChem Viewer accepts two types of molecular-data tabular input files. First, the user can specify a file where each data point is separated by a comma, with comma-containing entries placed in quotes (Table 1). This is the standard comma-separated-values (CSV) format used by many programs such as Microsoft Excel. Second, the user can specify a file where each data entry is separated by a tab character (i.e., tab-delimited, Table 1). We’ve found that we often generate tab-delimited files when using the Unix *paste* command.

**Table 1:** Examples of the program input files

**Format**	**Example**				
CSV (Excel)	ID Number, SMILES, Docking, Source, Synthesis Method				
	PLZKL-OMM-712, c1ccccc1, -5.2, "Pilzukil, Pharmaceuticals", Click Chemistry				
	PLZKL-OMM-677, CCOC, -9.3, Owen Moore Monet Pharma, Natural Product				
Tab delimited	ID Number	SMILES	Docking	Source	Synthesis Method
	PLZKL-OMM-712	c1ccccc1	-5.2	Pilzukil Pharmaceuticals	Click Chemistry
	PLZKL-OMM-677	CCOC	-9.3	Owen Moore Monet Pharm	Natural Product

Both comma-separated values (CSV) and tab-delimited data are accepted. The first row must contain data labels. Subsequent rows contain the data associated with each molecule.

Regardless of the specific format used, the first row of the input file must contain column labels, and subsequent rows must contain data listed in the same order. Each row represents a single molecule; row entries might include any molecule-associated data, such as the molecular name, weight, SMILES string, or a docking score.

Next, the user provides WebChem Viewer with information about the input data set. First, the user indicates which of the data columns contains the required SMILES strings, from which two-dimensional representations of each molecule are automatically generated. The user next indicates the column to use to initially sort the data, either in ascending or descending order. For example, we often wish to communicate the results of our virtual screens with collaborators. The docking scores associated with each molecule attempt to predict the free energy of binding; consequently, more negative scores represent better candidate ligands. A reasonable initial sorting, then, might be to order the data by the docking score, ascending from the lowest (most negative) value to the highest (least negative).

Finally, the user can specify which columns to initially hide. In our experience, it is often helpful to include supplementary information about each molecule that is useful but not critical for understanding. This data can be initially hidden and subsequently viewed by collaborators only when specifically requested. For example, in presenting the results of a virtual screen, the molecule name, structure, and docking score are clearly paramount. Associated data like the number of Lipinski violations [7] are useful but not necessarily critical, and so might be initially hidden.

### WebChem viewer output/portability

WebChem Viewer produces output that is HTML formatted. HTML is the same format used to create internet web pages; consequently, the output can be viewed in any modern web browser, on any computer operating system (including mobile devices). In our experience, this degree of portability is critical for may projects. For example, computational chemists almost exclusively use Unix-based operating systems, while most other researchers use Windows. Those research groups that have their own systems for sharing sets of molecular data (and many do not) typically resort to programs that are expensive, excessively complex, or operating-system dependent. It is not always practical to tell collaborators that they need to buy PerkinElmer’s ChemDraw or download Schrödinger’s Maestro, especially given that these packages include many features not required for simple data sharing. By divorcing data sharing from any specific operating system or computer program and instead wedding it to the ubiquitous web browser, collaborators need only click an html file sent by email in order to visualize the data immediately.

These challenges are hardly unique to scientific sharing. They are the very issues driving the current broader interest in “cloud computing” (i.e., deploying desktop or mobile apps through the internet rather than through the operating system). Given the recent proliferation of available operating systems (Android, iOS, Mac OSX, Windows, Linux, etc.) and the potentially imminent ascent of a number of others (e.g., Chrome and Firefox OS), operating-system independence is more critical than ever.

Given that WebChem Viewer’s output is so inherently portable, it is ideally suited for incorporation into existing cloud-based chemistry applications. As we expect most users will use WebChem Viewer to simply share data sets with colleagues, by default the program generates a single HTML file with all the required dependencies (javascript, css style-sheets, and image files) embedded directly into the code. However, WebChem Viewer can also save its HTML and dependencies as separate files so that the relevant portions of the output can be more easily incorporated into existing web-app frameworks. Indeed, in collaboration with the National Biomedical Computation Resource, we are currently pursuing plans to incorporate WebChem Viewer into a number of online chemistry applications. We are hopeful that other organizations will similarly find that WebChem Viewer satisfies their cloud-based project needs.

### Smiley2png 1.0 opal service

If required, users and software developers are also invited to access the remote Smiley2png 1.0 Opal service that permits WebChem Viewer to generate images of molecular structures “in the cloud,” either directly through the National Biomedical Computation Resource’s Opal Dashboard or programmatically through an Opal client (https://sourceforge.net/projects/opaltoolkit/files/opal-python/). The web interface accepts three parameters. First, the user must provide a text file, where each line represents a single compound and includes the name of the image file to generate as well as the associated SMILES string, separated by a space. Additionally, the user can specify the size of the square PNG image to generate, in pixels, as well as an email address for job-completion notification.

### Comparing WebChem viewer to existing software packages

WebChem Viewer has a number of advantages over existing packages. 1) It is simple and easy to use because it is singularly dedicated to generating easy-to-interpret reports. The user need only provide the data set, specify a few output options, and click the “Run” button to generate highly portable output files. 2) It is free for all users, including researchers working in industry, and is entirely open source so that knowledgeable users can modify the program according to their needs. 3) The program itself requires only a python interpreter and so can run on all modern desktop operating systems. 4) The output can be viewed in any web browser, including on mobile devices, without the need for additional plugins/programs. 4) The output is HTML formatted and so can be easily incorporated into existing websites to allow even broader data sharing if required.

Table 2 demonstrates how WebChem Viewer compares to Schrödinger’s Maestro Suite [8], PerkinElmer’s ChemDraw [9], and ChemAxon’s Instant JChem, three popular chemical-database management packages. Like WebChem Viewer, all three of these programs have Graphical User Interfaces that allow the user to generate tabular chemical reports that include both structural images and associated molecular information. However, WebChem Viewer has a number of advantages that are worthy of mention.

**Table 2:** Software comparison

**Name**	**Simplicity/usability**	**Programs required to view the output**	**Cost/open source**	**Operating systems**
WebChem Viewer	A single program dedicated only to generating reports.	Any modern web browser	Free for everyone. Open source.	Linux, OSX, Windows
Maestro	“The unified interface for all Schrödinger software,” with advanced tools to support molecular modeling, drug discovery, etc.	Maestro	Free version available. Closed source.	Linux, older versions of OSX, Windows.
ChemDraw with Microsoft Excel	A full featured chemical drawing program with myriad tools for conversion, querying, enumerating, etc.	ChemDraw, Microsoft Excel	Commercial license: $540-$1,540. Closed source.	Windows only
Instant JChem	Straightforward interface for managing molecular databases.	Instant JChem, Adobe Acrobat Reader, Microsoft Excel	Free for academics, $420-$1610 otherwise. Closed source.	Linux, OSX, Windows

WebChem Viewer compared to Schrödinger’s Maestro, PerkinElmer’s ChemDraw, and ChemAxon’s Instant JChem.

Schrödinger’s closed-source Maestro Suite, the “unified interface for all Schrödinger software,” includes many useful tools that facilitate computer-aided drug discovery and computational biology generally. However, when one wants only to generate a simple report to share with collaborators, these additional features adversely impact simplicity and usability. Maestro’s complexity aside, Schrödinger’s free version is capable of producing tabular reports that include images of chemical structures. If collaborators are willing to download Maestro as well (1.4 GB as of 11/2013), they can search and sort any Maestro-formatted molecular data shared with them. Maestro runs under Linux, Windows, and older versions of OSX, but is not currently compatible with OSX 10.9; furthermore, Maestro-formatted data files cannot be viewed on mobile devices or easily incorporated into existing web pages. Given WebChem Viewer’s simplicity and portability, we believe it is better suited for the singular task of generating simple compound-library reports.

PerkinElmer’s ChemDraw is a chemical drawing program with myriad tools for conversion, enumeration, querying, etc. These features, while useful in many contexts, again adversely impact simplicity and usability when one wishes only to generate a simple report. Furthermore, ChemDraw is closed source and costly (commercial licenses range from $540 to $1,540) and can only organize associated data in a tabular format via a Microsoft Excel plugin. Neither ChemDraw nor the Excel plugin run under Linux, and the plugin is incompatible with OSX as well. Furthermore, there is no easy mechanism for incorporating the output into existing web pages for broad data sharing.

Finally, ChemAxon’s Instant JChem, like WebChem Viewer, is singularly focused on displaying and organizing the contents of molecular databases. Unlike WebChem Viewer, however, Instant JChem is closed source and expensive ($420-$1,610 depending on the license), though ChemAxon does provide a free version for academics and a free viewer for all researchers. Instant JChem can also export molecular data sets to PDF and Microsoft Excel files for collaborators who don’t wish to download ChemAxon’s software. Like WebChem Viewer, Instant JChem runs on all modern desktop operating systems, and its PDF-formatted output is highly portable.

Furthermore, recognizing the modern importance of being able to share molecular data over the web, ChemAxon has also developed a version of Instant JChem that runs through Java Web Start. While the Web-Start version has its utility, WebChem Viewer’s HTML-formatted output is even better suited for web sharing. Due to recent Java security vulnerabilities, many users cannot run Java applets in their web browsers. For example, an analysis of user data collected from 17,514 people who visited the author’s personal website over the course of a recent month suggests that only 69% had browsers with Java enabled. In contrast, WebChem Viewer’s output does not require a Java installation.

### Distributed drug discovery: a test case

To verify WebChem Viewer’s utility in a real-life test situation, we recently used the program to further collaborations with the Distributed Drug Discovery (D3) initiative [10-12]. D3 is an educational initiative that allows undergraduate students to generate and test chemical compounds that could one day be developed into new drugs. Our efforts have focused on using computer-aided drug-design techniques to guide future student synthesis. In this context, we’ve needed to share large amounts of chemical data with D3 collaborators.

WebChem Viewer has greatly facilitated D3-based efforts. Our collaborators have specifically commented on the utility of the sorting, searching, and column-hiding features. Additionally, because WebChem-Viewer output is HTML formatted, we have been able to modify its appearance according to our collaborators’ requests.

In the past, we used Microsoft Excel to tabulate our data when sharing with collaborators. As our particular operating system is not compatible with ChemDraw’s Excel plugin, we were forced to manually convert individual SMILES strings to images on a compound-by-compound basis and to tediously copy the resulting images from ChemDraw into Excel. As good collaborations often involve back-and-forth feedback, a given project frequently required us to repeat this process many times as we modified our computational protocols in response to collaborators’ suggestions.

In contrast, with WebChem Viewer the D3 collaboration has been streamlined. Structural images are incorporated into the reports automatically, thus lowering the barrier required to implement new suggestions. These benefits were obtained without requiring our collaborators, who are not computationalists, to install additional software.

### Stability

To test the stability of WebChem Viewer, we first obtained a list of 162,161 SMILES strings by downloading and processing the “Clean Fragments” subset of the ZINC database [13]. We then generated 200 tabular input files by randomly selecting 15 SMILES strings per file and associating 5 to 10 dummy variables with each compound. These dummy variables consisted of randomly chosen numbers ranging from 0 to 100 and/or randomly generated text sequences of 10 letters. WebChem Viewer processed the first 100 input files using a local copy of Open Babel to generate molecular-structure images. The last 100 input files were similarly processed, except the remote image-generating server (“Smiley2png”) was employed. In all cases, WebChem Viewer produced the appropriate output files without any errors.

## Conclusions

WebChem Viewer provides a simple and free way to share substantial quantities of heterogeneous chemical data. The program has been specifically tested on a number of operating systems, using several different versions of Open Babel and Python (Table 3). Additionally, WebChem-Viewer output has been successfully visualized in a number of web browsers (Table 4). Sample data sets in both the CSV and tab-delimited formats is provided with the download so that interested users can easily experiment with the program. Tutorials are also included in the Supporting Information (Additional files 1 and 2). We are hopeful that both WebChem Viewer as well as its associated web application for generating images of molecular structures will be useful tools for computational and medicinal chemists, as well as their collaborators.

**Table 3:** Webchem viewer operating-system compatibility

**Operating system**	**Open babel version**	**Python version**
Scientific Linux 6.2	2.3.1	2.6.6
Mac OS X 10.8.3	2.3.1	2.7.2
Windows XP Professional	2.3.2	2.5
Windows XP Professional	2.3.2	2.6
Windows XP Professional	2.3.2	2.7.3

WebChem Viewer has been tested on a number of operating systems, with various Open-Babel and Python versions. We note that most users will not need to download and install Open Babel on their own machines.

**Table 4:** Webchem viewer browser compatibility

**Operating system**	**Web browser**
Scientific Linux 6.2	Chrome 26.0.1410.63
Scientific Linux 6.2	Firefox ESR 17.0.5
Mac OS X 10.8.3	Chrome 29.0.1547.32 beta
Mac OS X 10.8.3	Firefox 22.0
Mac OS X 10.8.3	Safari 6.0.4
Android 4.1.2 (Tablet)	Chrome 28
Android 4.1.2 (Tablet)	Default Android Browser (as of 8/2013)
iOS 6.1.3 (Tablet)	Mobile Safari 6
Windows XP Professional	Firefox 3.0.6
Windows XP Professional	Chrome 28.0.1500.95 m
Windows XP Professional	Internet Explorer 8.0.6001.18702
Windows 7	Internet Explorer 10.0.9200.16635

WebChem Viewer’s HTML output files have been successfully visualized on many web browsers, running under several operating systems.

## Availability and requirements

**Project name:** WebChem Viewer

**Project home page:**http://nbcr.ucsd.edu/WebChemViewer

**Operating systems:** Platform independent

**Programming language:** Python, HTML, JavaScript

**Other requirements:** Python 2.x (tested on versions 2.5 and higher), Open Babel (optional if the user wants to generate molecular images locally rather than using our sever application; tested on versions 2.3.1 and 2.3.2)

**License:** FreeBSD license

**Any restrictions to use by non-academics:** None

## Competing interests

The authors declare that they have no competing interests.

## Authors’ contributions

JDD served as chief programmer and drafted the initial version of the manuscript. REA critically revised the manuscript for important intellectual content. Both authors read and approved the final manuscript.

## Supplementary Material

Additional file 1 WebChem Viewer Tutorial. Click here for file

Additional file 2 Smiley2png 1.0 Tutorial. Click here for file
