# Supplementary material for: WebChem Viewer: a tool for the easy dissemination of chemical and structural data sets
Source: BMC Bioinformatics. 2014 May 23;15:159. doi: 10.1186/1471-2105-15-159 (PMC4094277; doi:10.1186/1471-2105-15-159)

WebChem Viewer Tutorial

(A video tutorial is available at https://vimeo.com/81233812)

Step 1. Compile your molecular data using a program like Microsoft Excel. One of the columns should include the SMILES strings describing the molecular structures.

	A	B	C	D	E
1	ID Number	SMILES	Docking Score	Source	Synthesis Method
2	PLZKL-OMM-71287	c1ccccc1[C@@H]([C@@H](O)C(=O)N[C@@H](C([O-])=O)C)NC(=O)c2ccccc2	-5.2	Pilzukil Pharmaceuticals	Click Chemistry
3	PLZKL-OMM-67790	CCOC(=O)C[C@H](C(=O)OCC)[C@H](C([O-])=O)NC(=O)c1c(Cl)cccc1	-9.3	Owen Moore Monet Pharmaceuticals	Natural Product
4	PLZKL-OMM-67790	CCOC(=O)C[C@H](C(=O)OCC)[C@H](C([O-])=O)NC(=O)c1c(Cl)cccc1	-11.6	Owen Moore Monet Pharmaceuticals	Natural Product
5	PLZKL-OMM-67790	CCOC(=O)C[C@H](C(=O)OCC)[C@H](C([O-])=O)NC(=O)c1c(Cl)cccc1	-3.6	Pilzukil Pharmaceuticals	Click Chemistry
6	PLZKL-OMM-67790	CCOC(=O)C[C@H](C(=O)OCC)[C@H](C([O-])=O)NC(=O)c1c(Cl)cccc1	-7.2	Pilzukil Pharmaceuticals	Natural Product
7	PLZKL-OMM-71297	CC(C)[C@H](C([O-])=O)NC(=O)[C@H](O)[C@H](c1ccccc1)NC(=O)c2ccccc2	-8.9	Owen Moore Monet Pharmaceuticals	Natural Product
8	PLZKL-OMM-83542	c1cccc(I)c1C[C@@H](C([O-])=O)NC(=O)[C@H](O)[C@H](c2ccccc2)NC(=O)c3ccccc3	-10.5	Pilzukil Pharmaceuticals	Click Chemistry
9	PLZKL-OMM-83508	C1CC1C[C@@H](C([O-])=O)NC(=O)[C@H](O)[C@H](c2ccccc2)NC(=O)c3ccccc3	-7.2	Owen Moore Monet Pharmaceuticals	Natural Product
10	PLZKL-OMM-83491	CC[C@@H](C([O-])=O)NC(=O)[C@H](O)[C@H](c1ccccc1)NC(=O)c2ccccc2	-14.9	Pilzukil Pharmaceuticals	Click Chemistry
11	PLZKL-OMM-83490	c1ccccc1[C@@H]([C@@H](O)C(=O)N[C@H](C([O-])=O)C)NC(=O)c2ccccc2	-6.3	Pilzukil Pharmaceuticals	Click Chemistry

Step 2. Save this data in the CSV format.

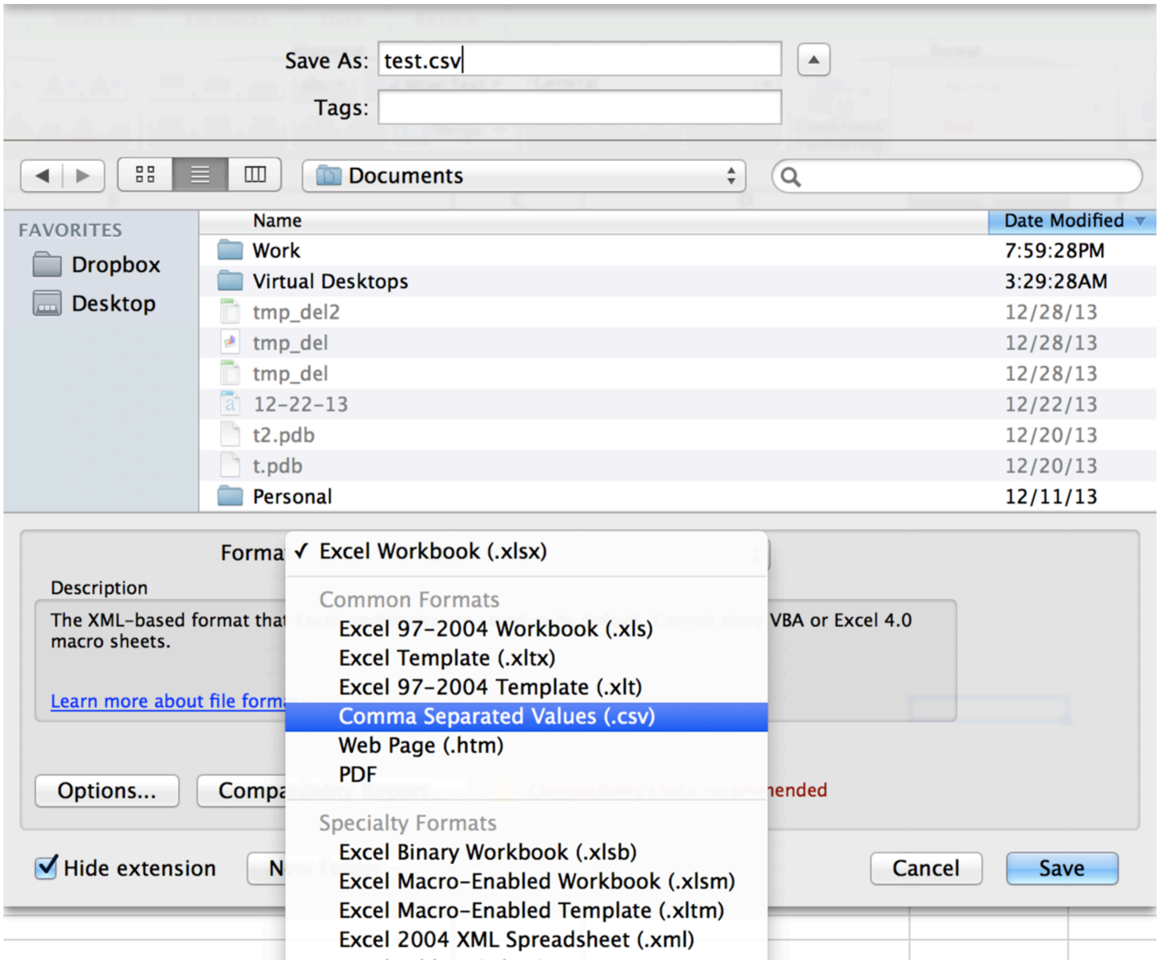

Step 3. Download WebChem Viewer and uncompress the ZIP file. Run the appropriate version of the program for your operating system.

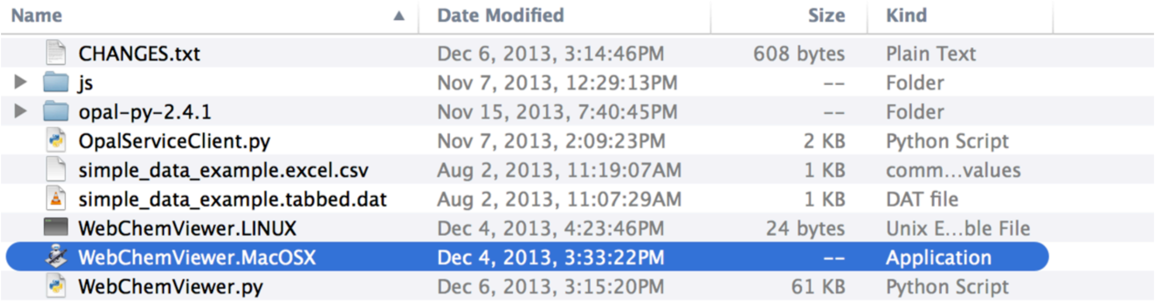

Name	Date Modified	Size	Kind
CHANGES.txt	Dec 6, 2013, 3:14:46PM	608 bytes	Plain Text
js	Nov 7, 2013, 12:29:13PM	--	Folder
opal-py-2.4.1	Nov 15, 2013, 7:40:45PM	--	Folder
OpalServiceClient.py	Nov 7, 2013, 2:09:23PM	2 KB	Python Script
simple_data_example.excel.csv	Aug 2, 2013, 11:19:07AM	1 KB	comm…values
simple_data_example.tabbed.dat	Aug 2, 2013, 11:07:29AM	1 KB	DAT file
WebChemViewer.LINUX	Dec 4, 2013, 4:23:46PM	24 bytes	Unix E…ble File
WebChemViewer.MacOSX	Dec 4, 2013, 3:33:22PM	--	Application
WebChemViewer.py	Dec 6, 2013, 3:15:20PM	61 KB	Python Script

Step 4. Select the test.csv file you saved form Excel. Since it's a CSV file, click on the "Comma-Separated Values" option, and then "Load Data".

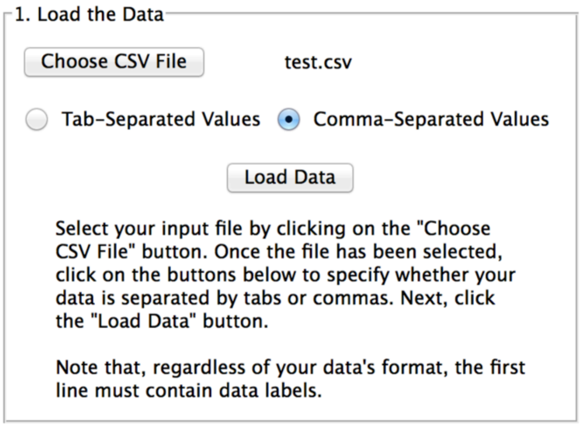

Step 5. Specify the SMILES column, the sort column, and the sort order. Here, I want to initially sort the data by the Docking Score. Since lower docking scores represent better binders, I will sort the scores in ascending order.

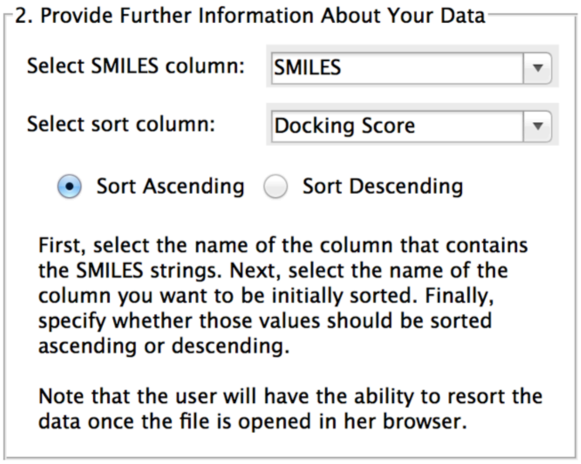

Step 6. If you have a working copy of Open Babel installed on your computer, use this button to specify the location of the obabel executable file. If you don't have Open Babel installed, just skip this step. WebChem Viewer will use your internet connection to contact a remote server where images of your molecule will be generated "in the cloud."

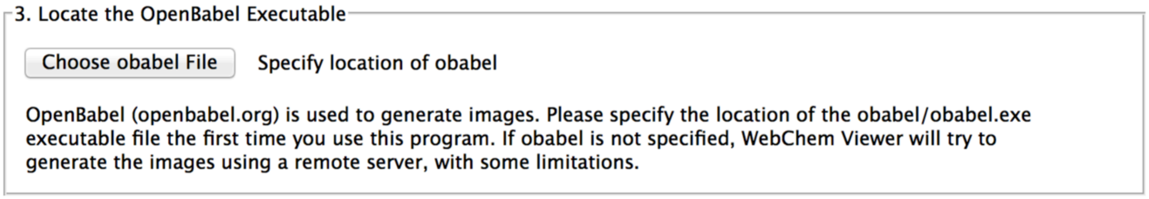

Step 7. Provide additional output options. Most users will want to save the output as a single HTML file. Here, I've chosen to name my file "test.html". I've also indicated that the SMILES and Source data should be initially hidden. Once ready, go ahead and click on the "Create File!" button.

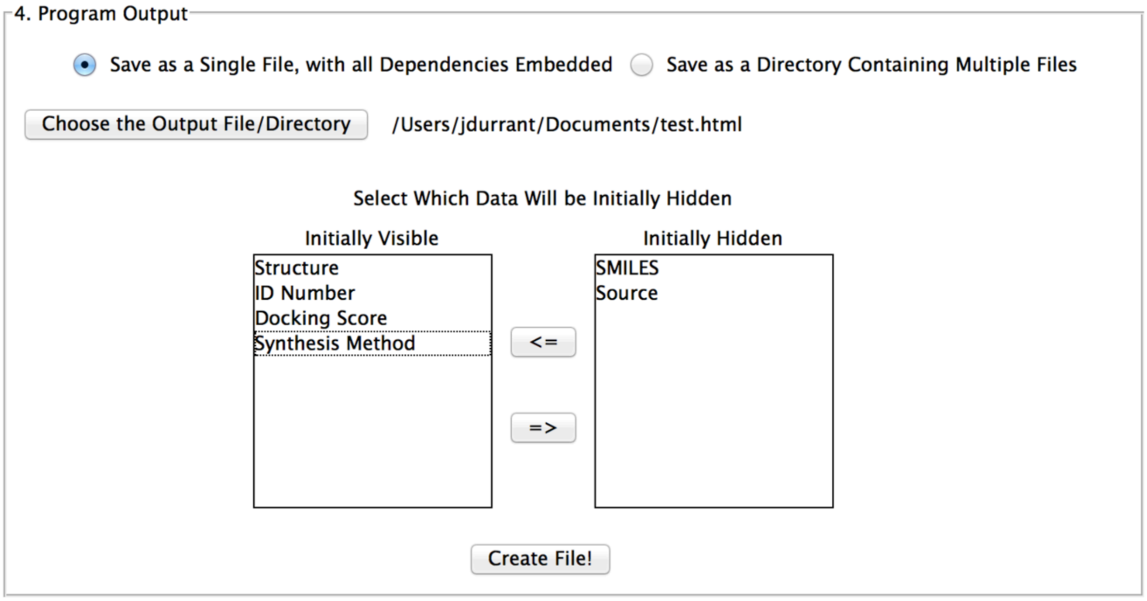

Step 8. You and/or your collaborators can view the output in any web browser. For example, in Google Chrome, select the File ➔ Open File… option.

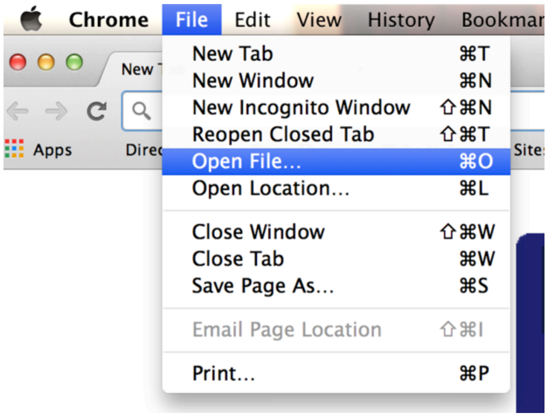

Step 9. Then select the HMTL file you generated using WebChem Viewer.

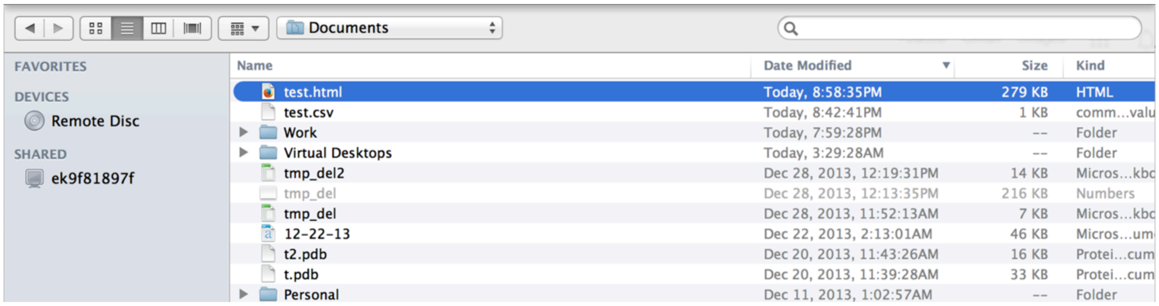

Step 10. Here's what the output looks like. The HTML file can be sent by email to collaborators.

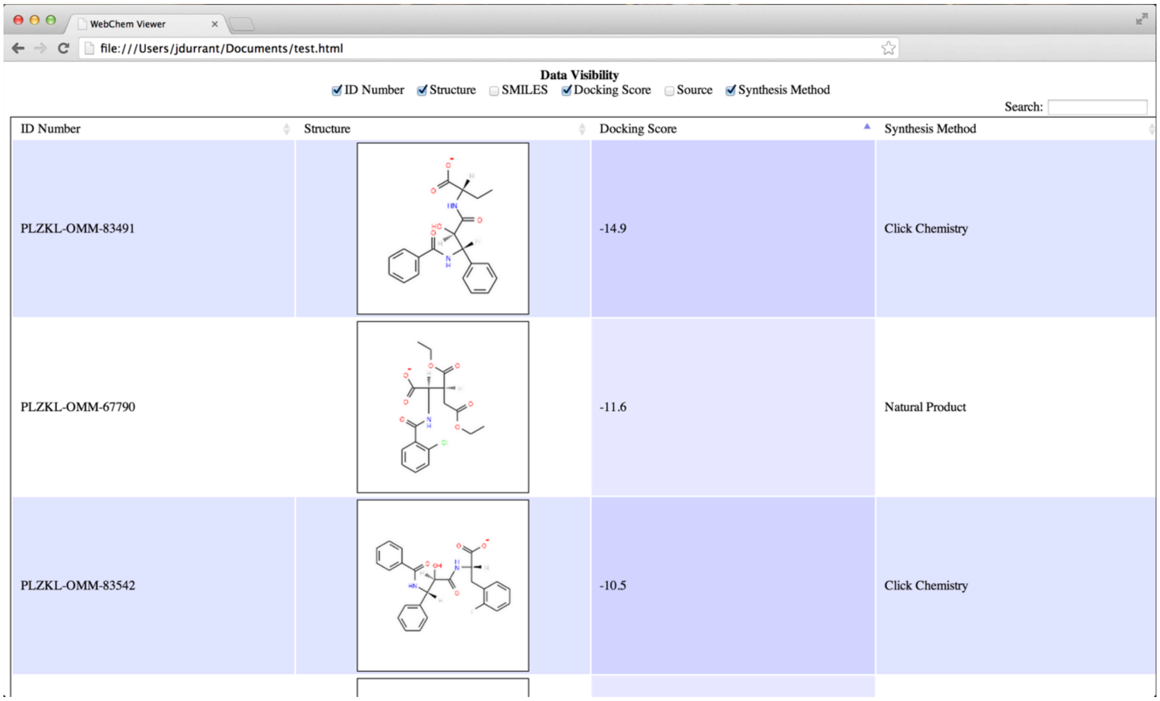

Supplement: Additional file 1 — WebChem Viewer Tutorial. [file 1471-2105-15-159-S1.pdf]
