# Supplementary material for: WebChem Viewer: a tool for the easy dissemination of chemical and structural data sets
Source: BMC Bioinformatics. 2014 May 23;15:159. doi: 10.1186/1471-2105-15-159 (PMC4094277; doi:10.1186/1471-2105-15-159)
Supplement: Additional file 2 — Smiley2png 1.0 Tutorial. [file 1471-2105-15-159-S2.pdf]

Smiley2png 1.0 Tutorial

Step 1. Start by going to the Opal Dashboard. Try this URL: http://nbcr-222.ucsd.edu/opal2/dashboard

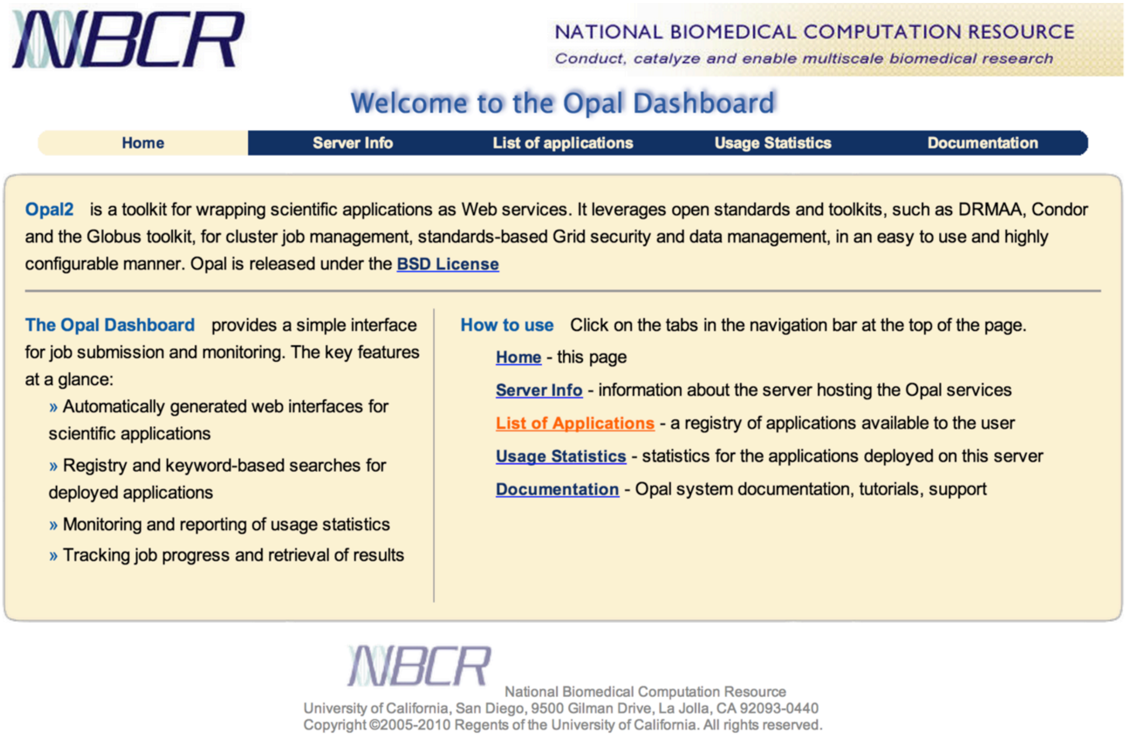

Click on the "List of applications" menu.

Step 2. Select the "Smiley2png 1.0" service. Use the link in orange to access the online submission system, or the link in blue for programmatic access. For the purposes of this tutorial, click on the orange link.

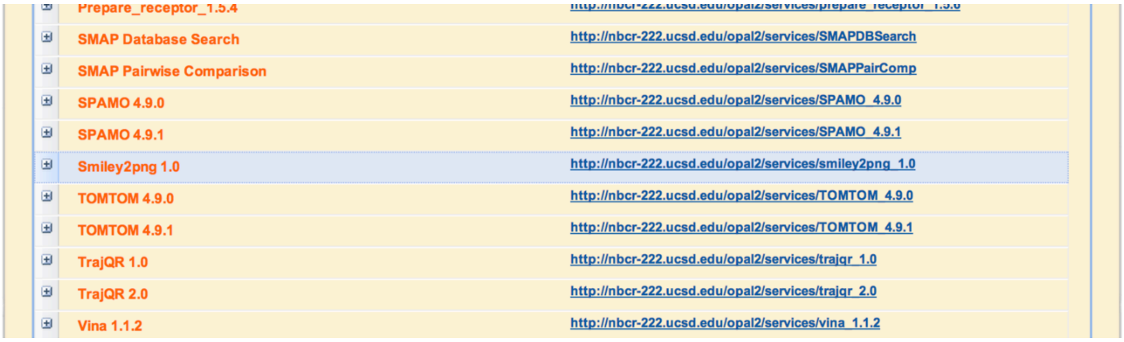

Step 3. Type in your email so the online system can notify you when your output is ready.

Insert user email for status notification:	some_email@myuniversity.edu

Step 4. Select your Smiley2png input file. This is a simple text file, where each line represents an individual molecule. The first item in each line is the name of the corresponding PNG file to generate. The second item is the SMILES string representing that molecular structure. These two items should be separated by a space.

Input file (see help for format description) *	Choose File molecular_data.dat

Simple text files can be generated using programs like Komodo Edit or Windows Notepad. For example, the contents of the text file could look like this:

```
img1.png CCCCCC
img2.png CCNCCC
img3.png CCCC=O
```

Step 5. Specify the dimensions of the PNG images files to generate. For example, if you use "200", the PNG files will be 200 x 200 pixels, square.

Output file geometry (an integer, for example 100)	200

Click the "Submit" button when you're done.

Step 6. Now wait for the remote servers to generate all the requested images. When you get an "Execution complete" message, click on the link to see your results.

Submission results for smiley2png_1.0

Date and time :	**1/1/2014 9:51:00 PM**
JobId :	appsmiley2png_1.01388638230264-1796498967
Status code:	8
Message:	Execution complete - check outputs to verify successful execution
Output Base URL:	http://nbcr-222.ucsd.edu/opal-jobs/appsmiley2png_1.01388638230264-1796498967

Step 7. The remote server will generate a number of files. You're interested in the one named "output-images.tar.gz". Download the file by clicking the appropriate link. Then, uncompress the file to view your PNG images.

Directory Listing For /appsmiley2png_1.01388638230264-1796498967/ - Up To /

Filename	Size	Last Modified
molecular_data.dat	0.1 kb	Thu, 02 Jan 2014 04:50:30 GMT
output-images.tar.gz	7.2 kb	Thu, 02 Jan 2014 04:50:46 GMT
sge.sub	0.2 kb	Thu, 02 Jan 2014 04:50:30 GMT
smi2png-err	0.1 kb	Thu, 02 Jan 2014 04:50:46 GMT
smi2png-out	0.1 kb	Thu, 02 Jan 2014 04:50:46 GMT
stderr.txt	0.0 kb	Thu, 02 Jan 2014 04:50:30 GMT
stdout.txt	0.1 kb	Thu, 02 Jan 2014 04:50:46 GMT
vinput	0.1 kb	Thu, 02 Jan 2014 04:50:30 GMT

Apache Tomcat/5.5.17
